# Psychosocial work environment beyond WEIRD: meta-analytic and psychometric evidence on the Job Content Questionnaire

**DOI:** 10.3389/fpsyg.2025.1642607

**Published:** 2025-10-02

**Authors:** Adrián García-Selva, Beatriz Martín-del-Río, Marcelo Leiva-Bianchi

**Affiliations:** ^1^Department of Behavioural Sciences and Health, University of Miguel Hernández, Elche, Spain; ^2^Laboratory of Methodology, Behaviour and Neuroscience, Faculty of Psychology, University of Talca, Talca, Chile

**Keywords:** demand-control model, job strain, job control, psychological demands, validation, meta-analysis

## Abstract

**Background:**

The psychosocial work environment significantly impacts employee well-being and performance. Among the most recognized models for assessing psychosocial risk factors is the Job Demand-Control-Support (JDCS) model, which posits that psychological demands, job control, and social support are core determinants of work-related stress. Although extensively studied, research on its measurement tools—particularly the Job Content Questionnaire (JCQ)—has been disproportionately conducted in WEIRD countries, raising questions about cross-cultural validity.

**Objective:**

This study aimed to (I) evaluate the reliability of JCQ dimensions across cultures through a meta-analytic approach and (II) validate a 15-item short version of the JCQ in a large and culturally distinctive Spanish sample.

**Methods:**

A meta-analysis of 21 studies (*N* = 21,732) from WEIRD and non-WEIRD countries assessed the internal consistency of psychological demands and job control dimensions. Additionally, an empirical validation was conducted with 860 Spanish workers using exploratory structural equation modeling (ESEM) to test factorial structure, reliability, and measurement invariance across gender, job level, and educational background.

**Results:**

Meta-analytic results showed moderate to high internal consistency for job control (*α* = 0.737) and psychological demands (α = 0.603), with higher reliability in WEIRD populations for job control. The Spanish validation supported a four-factor ESEM model with excellent fit and invariance across demographic groups. All dimensions showed strong composite reliability and convergent validity.

**Conclusion:**

This research confirms the robustness of the JCQ’s core constructs and supports the use of a concise, psychometrically sound version of the instrument across diverse sociocultural contexts. It also advances equitable psychometric practices by bridging WEIRD and non-WEIRD research efforts.

## 1 Introduction

The psychosocial work environment is a critical determinant of employee well-being, productivity, and overall health (Vanroelen et al., 2021). Currently, employee well-being is not just a health issue for companies. There is growing evidence that caring for and improving people’s occupational health leads to greater development and benefits for their organizations (Lari, 2024). In this regard, the identification and early intervention of psychosocial factors that predispose workers to occupational stress has become extremely important and is considered a key strategy in the organizational environment, as it not only promotes the well-being of professionals and prevents the onset of job stress, but also helps organizations achieve and maintain an adequate level of productivity and efficiency (Gonçalves et al., 2022).

At this point, it is crucial to distinguish between psychosocial factors and psychosocial risk factors. Psychosocial factors refer broadly to all aspects of the design, organization, and management of work, as well as their social and organizational contexts. These factors are not inherently negative; for example, a manageable workload or autonomy can be beneficial. However, they become psychosocial risk factors when they have a high probability of negatively affecting the health and well-being of workers, such as an excessive workload, tight deadlines, lack of role clarity, or low control over tasks (Fernandes and Pereira, 2016). Therefore, identification and management strategies in occupational health focus on mitigating the latter, transforming them into conditions that promote well-being.

Among the most influential theoretical frameworks for understanding these psychosocial risk factors is the Job Demand-Control-Support model (Karasek, 1979; Johnson and Hall, 1988). Originally, Robert Karasek (1979) introduced the Job Demand-Control model to explain how job strain, a primary precursor to work-related stress and ill-health, arises from the interaction of high psychological demands and low job control. Within this model, psychological demands refer not only to the amount of work to be done (quantitative demands) such as overload or time pressure, but also encompass qualitative aspects, such as the need to hide emotions, the complexity of tasks, or making difficult and quick decisions. For its part, job control is a two-dimensional construct that includes, on one hand, decision authority, which is the worker’s ability to make decisions about their own work; and on the other, skill discretion, which refers to the opportunity to use and develop one’s own skills and creativity in tasks, as opposed to repetitive and unchallenging work (Karasek and Theorell, 1990). Later, social support was integrated into this model as a relevant characteristic of the work environment, becoming known as the Job Demand-Control-Support model (Johnson and Hall, 1988; Johnson et al., 1989). This new factor refers to the support provided by the hierarchy and colleagues in the workplace, and is considered a possible resource to moderate the stress generated by the combination of high demands and low control. The simplicity of the model and its practical application have made this theoretical framework one of the most influential in the study of the psychosocial work environment and its relationship to occupational health (Dutheil et al., 2022).

Therefore, accurately measuring these dimensions is critical for research, organizational interventions, and public health policy. The Job Content Questionnaire (JCQ) (Karasek et al., 1998) has historically been a cornerstone instrument for operationalizing these constructs, utilized extensively across diverse occupational sectors and national contexts. Considerable research (e.g., Fransson et al., 2012; Formazin et al., 2025; Karasek et al., 2007; Mutambudzi et al., 2019), has shed light on the measurement properties of various JCQ versions. These studies generally indicate consistent psychometric characteristics for its core dimensions, particularly psychological demands and job control, which tend to show robust convergent validity across different instrument formats and satisfactory internal consistency in varied samples. This body of work supports their utility in assessing key aspects of the work environment.

In addition to the Job Content Questionnaire, several other instruments have been developed to assess psychosocial risk factors at work. Among the most frequently cited are the Copenhagen Psychosocial Questionnaire (COPSOQ) (Kristensen et al., 2005), and the Effort-Reward Imbalance (ERI) model questionnaire (Siegrist, 1996). Each tool conceptualizes and measures workplace psychosocial risk factors differently: for example, the COPSOQ provides a broader assessment including organizational and social dimensions, while the ERI focuses on the perceived imbalance between efforts and rewards. These instruments have demonstrated acceptable psychometric properties, with studies frequently reporting satisfactory internal consistency and evidence of construct validity in diverse occupational contexts (Berthelsen et al., 2018; Stanhope, 2017; Useche et al., 2019). Their development and use have contributed significantly to advancing research and practice in occupational health, offering complementary approaches to understanding psychosocial risks factors at work.

However, the landscape of psychological assessment, especially concerning well-being, has been predominantly shaped by research in Western, Educated, Industrialized, Rich, and Democratic (WEIRD) societies (Wooliscroft and Ko, 2023). It is relevant to note that the measurement of constructs such as psychological demands and job control has followed an uneven historical and geographical trajectory. Systematic interest in these factors emerged prominently in WEIRD countries, particularly in Scandinavia and North America, during the 1980s, driven by a paradigm shift in occupational health that moved from an exclusive focus on physical risks to one that included psychosocial ones. Within this context, the Job Demand–Control model and its core dimensions were first empirically evaluated in WEIRD settings. Karasek et al. (1998), for instance, tested the JCQ in large samples from the United States, Canada, and the Netherlands, demonstrating robust cross-national reliability and structural similarity. In contrast, in many non-WEIRD nations, the integration of psychosocial risk assessment into occupational health and safety policies occurred later, as prevention systems often prioritized physical and chemical hazards, reflecting different stages of industrial development and legislative priorities (Jain et al., 2011). Nevertheless, early validations in non-WEIRD contexts soon followed. Further adaptations appeared during the 2000s in developing regions, including Brazil, Thailand, China, and Iran (e.g., de Araújo and Karasek, 2008; Phakthongsuk and Apakupakul, 2008; Li et al., 2004; Tabatabaee et al., 2013). Although these studies demonstrated the applicability of the JDC model in diverse cultural contexts, they were fewer in number and often revealed psychometric challenges, underscoring the need for more comprehensive and culturally nuanced validation efforts.

More specifically, a recent meta-analysis of the Job Demand-Control model (Pletzer et al., 2024) found that 56.7% of included studies were conducted in WEIRD countries (e.g., the United States, Germany, Australia), while the remaining 43.3% came from non-WEIRD settings (e.g., China, Turkey, Chile). This concentration is not unique to instruments measuring the JDC model. Research applying other prominent questionnaires, such as the COPSOQ and the ERI questionnaire, has also been disproportionately centered in WEIRD countries. Studies from non-WEIRD settings, while less common, often highlight significant cultural and linguistic challenges in achieving conceptual equivalence during their adaptation processes in regions like Latin America (Juárez-García et al., 2015; Gonçalves et al., 2021) or Asia (Eum et al., 2007a; Li et al., 2012).

As pointed by some authors (Cornesse and Bosnjak, 2018; Wild et al., 2022), constructs related to well-being and perceived health can be highly dependent on cultural context, so the greater presence of research conducted in WEIRD countries raises concerns about possible biases and difficulties in generalizing. This call emphasizes that validity is not an inherent property of a test but must be empirically demonstrated in each specific context of application (Cruchinho et al., 2024). At this point, there is a need to promote psychometric developments in non-WEIRD countries, which are often underrepresented despite hosting the majority of the world’s population (Beyebach et al., 2021).

In this line, more than a decade has passed since Fransson and collaborators published their paper “Comparison of alternative versions of the job demand-control scales in 17 European cohort studies: the IPD-Work consortium” (Fransson et al., 2012). This was the first study to measure, among other variables, job strain by comparing data obtained with European standards. The instrument chosen was the JCQ (Karasek et al., 1998), since it considers the empirical and psychometric development of Karasek’s two-dimensional model. Despite its quality, their studies used different dimensions or items of the JCQ. In addition, some of the studies reviewed used the Demand Control Questionnaire (DCQ) (Theorell et al., 1988; Theorell, 1996), an instrument that also measures job strain. Scales and dimensions were considered equivalent versions of JCQ because they measured the same dimensions: psychological demands and job control. As shown in Tables 3, 4 of Fransson et al. (2012) article, these versions strongly correlate, ranging from 0.759 to 0.984. This is an indicator of convergent validity with its criterion or gold standard: the JCQ total score. This study has made a fundamental contribution to examining the validity of job demand-control scales.

However, since the IPD-Work consortium primarily analysed data from European cohorts—thus reflecting largely WEIRD populations—there remains a gap in understanding how the JCQ performs across broader sociocultural and economic contexts. Given the growing attention to the cultural specificity of psychological constructs and the increasing recognition that findings derived from WEIRD countries may not generalize globally (Schimmelpfennig et al., 2024), it becomes essential to assess the global usage patterns and psychometric performance of the JCQ in both WEIRD and non-WEIRD contexts. For this reason, there is a need to evaluate the construct validity of psychological demands and job control of any of the multiple versions of the JCQ, whether or not they are included in the study by Fransson et al. (2012). This also implies the need to extend validation studies of different versions of the JCQ to more culturally diverse contexts than those traditionally used to explore the psychometric characteristics of this instrument. Therefore, this article aims to foster scientific collaboration between WEIRD and non-WEIRD countries, bringing together research teams from both contexts under shared objectives.

The present research aims to contribute to the ongoing discussion on the validity and reliability of the JCQ, with the objective of analysing the validity and reliability of the dimensions of the Job Demands-Control-Support model (Karasek, 1979; Johnson and Hall, 1988). For this purpose, (I) a systematic review of the mainstream literature will be conducted to identify additional studies reporting Cronbach’s *α* coefficients as indicators of the internal consistency of job demand-control scales, to be meta-analysed incorporating a differentiated analysis of studies conducted in WEIRD and non-WEIRD populations to enable cross-cultural comparisons of reliability evidence. Finally, (II) an empirical psychometric study will be carried out in a large Spanish sample to validate a short version of the JCQ, different from those applied by the IPD-Work consortium.

While Spain is traditionally considered a WEIRD country, its distinct cultural fabric, labor market dynamics, and socio-economic characteristics present a unique context compared to many North American or Northern European settings where the JCQ has been classically validated. The established robustness of the JCQ’s core constructs in well-studied WEIRD populations underscores the scientific value of exploring its psychometric performance in such differentiated settings. This exploration is crucial because cultural or structural differences can influence how questionnaire items are interpreted and how constructs manifest (Cruchinho et al., 2024). This highlights an ongoing need for rigorous primary validation studies that apply contemporary psychometric standards, particularly in populations that, while not strictly “non-WEIRD,” offer important socio-cultural diversity. The very consistency of findings for the JCQ’s core dimensions in traditionally studied populations reinforces the importance of broadening the research focus to ensure that such a widely utilized instrument maintains its conceptual and metric integrity, thereby enhancing both its local applicability and the global understanding of occupational stress.

## 2 Materials and methods

### 2.1 Participants

**Figure 1:**
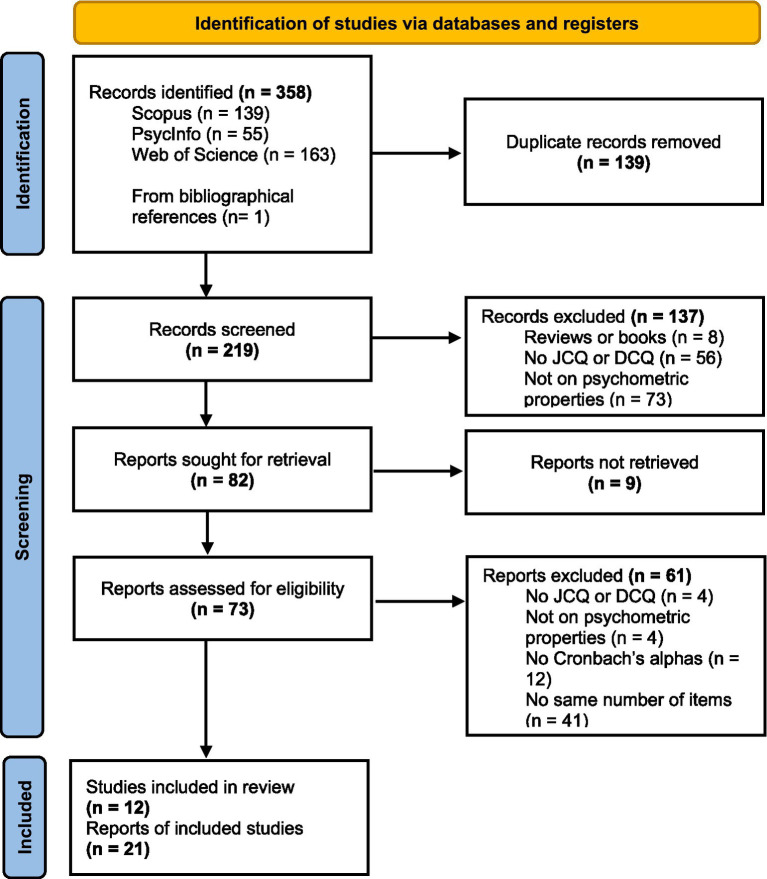
PRISMA flow chart.

For part (I), a systematic review and meta-analysis were carried out following PRISMA recommendations (Page et al., 2021). Searches were conducted in the WOS, Scopus and PsycINFO databases without language or date restriction until April 5, 2025, using the following search terms: (“job content questionnaire” OR JCQ OR “Demand Control Questionnaire” OR DCQ OR “Demand Control Support Questionnaire” OR DCSQ) AND (validity OR reliability OR “psychometric propert*” OR “internal consistency”) and adapting it to each search engine. Inclusion criteria were articles with primary research studies reporting on reliability indicators of the questionnaires in question. The exclusion criteria were as follows: (1) reviews or theoretical papers; (2) studies that did not use either of these questionnaires; (3) papers that did not address the psychometric properties of these instruments; (4) papers for which an adequate translation was not available; (5) papers that did not include Cronbach’s alpha values for the psychological demands and job control dimensions; and (6) papers with a different number of items in the psychological demands and job control (see Figure 1). Records and studies available in the databases were included through the institutional access provided by the universities to which the authors belong. Two reviewers independently examined the papers, and, in case of disagreement, a third reviewer was consulted. Thus, the reliability generalization meta-analysis was performed on 21 studies, published in 12 articles, including 21,732 participants from Thailand (*K* = 1), China (*K* = 2), Brazil (*K* = 2), Vietnam (*K* = 1), Greece (*K* = 1), Colombia (*K* = 3), USA (*K* = 2), Canada (*K* = 3), Netherlands (*K* = 1), Japan (*K* = 3), Iran (*K* = 1), and Korea (*K* = 1).

For part (II), the sample consisted of 860 individuals from the Spanish labor market, of which 451 (52.44%) were men and 409 (47.56%) were women. The average age of the total sample was 35.85 years (*SD* = 11.18), with a minimum of 18 and a maximum of 69. A total of 461 (53.60%) workers belonged to the basic level, while 241 (28.02%) were middle managers and 158 (18.38%) held executive positions. In terms of educational level, 309 (35.93%) participants completed secondary education, while 282 (32.79%) completed vocational training and 269 (31.28%) completed university studies. Finally, in terms of the distribution of the sample by employment sector, a total of 475 (55.23%) workers belonged to the service sector, while 110 (12.79%) belonged to industry, 80 (9.30%) were part of the education sector, 73 (8.49%) worked in public administration, 69 (8.02%) belonged to the commerce sector, and 53 (6.16%) worked in the healthcare sector.

### 2.2 Measures

For (I), reliability generalization meta-analysis was performed on the five-item versions of the psychological demands dimension and the nine-item versions of the job control dimension, which are common in the 21 systematically reviewed studies. For part (II), a 15-item questionnaire different from those applied by the IPD-Work consortium was used. This questionnaire was previously applied to hospital nurses (Evans and Steptoe, 2002), primary and high school teachers (Cropley et al., 1999; Steptoe et al., 2000) and the general population (Steptoe et al., 1996). This instrument is grouped into four dimensions, which correspond to psychological demands (items 4, 8, and 11), job control, divided into skill discretion (items 3, 7, 10, and 13) and decision authority (items 1, 6, and 15), and, finally, the dimension of social support (items 2, 5, 9, 12, and 14). Each item is rated on a 4-point Likert-type scale (1, strongly disagree; 4, strongly agree). The complete version of the questionnaire can be found in Supplementary materials.

### 2.3 Procedure

This study complied with the protocols of the ethics committee accredited by the Office of Responsible Research of the Miguel Hernández University (Reference DCC.ASP.01.20).

For the data collection process, the research team utilized online questionnaires created using Google Forms. These surveys were distributed to a broad spectrum of companies across various regions of Spain to capture a diverse sample of industries and organizations. Communication was initiated with HR managers and company directors, who assisted in disseminating the questionnaire among their staff. To ensure the standardization of the administration for each individual, the first page of the survey provided clear instructions regarding the study’s objective, the voluntary and anonymous nature of participation, an estimate of the completion time, and a contact email for any inquiries. This allowed participants to complete the questionnaire at their own convenience in an environment of their choosing. The inclusion criteria required participants to be of legal age (18 years or more) and actively employed at the time of data collection. No specific exclusion criteria were applied beyond these requirements. The data gathering took place in an organized fashion between 2022 and 2024.

### 2.4 Data analysis

For (I), meta-analyses were initially performed using Jamovi, while meta-regressions were conducted in Python. Unlike Jamovi, Python allowed for the inclusion of categorical moderators, such as WEIRD country status. Python provided extended statistical outputs (statsmodels and pandas libraries) and visualizations (matplotlib).

All meta-analyses considered random-effects models. All models were estimated using the DerSimonian-Laird method. Heterogeneity indices (*I^2^*; *Q*; *p* > 0.05) were calculated (Huedo-Medina et al., 2006). A random-effects model robust to non-compliance with the homogeneity assumption will be used (Botella and Sánchez-Meca, 2015; Hedges and Olkin, 1985). Models were estimated by the restricted maximum-likelihood method. The raw correlation/reliability coefficients were used. Meta-regressions were conducted using weighted least squares (WLS), incorporating mediators such as WEIRD country classification and percentage of women in the sample. Forest and meta-regression plots were generated, only if its results were significant. Publication bias was assessed through Egger’s regression and Rosenthal’s Fail-safe number (*N*_Rosenthal_ > 5*K + 10; *p* < 0.05).

For part (II), to validate the 15-item scale, the guidelines suggested by Ferrando et al. (2022) and Hernández et al. (2020) followed. To enhance the validity and the generalizability of the findings, a cross-validation approach was employed by randomly splitting the overall sample into two independent subsamples. To ensure that the two random subsamples were homogeneous, a chi-square statistical test was performed between the sociodemographic variables to explore possible significant differences in both subsamples. Finally, before beginning the analyses proposed below, reverse items of the questionnaire (7 and 14) were recoded.

The next step was to perform an exploratory factor analysis (EFA) using the first subsample (*n*_1_ = 429) to examine the dimensional structure of the scale. Before proceeding, the suitability of the data for factor analysis was assessed through the Kaiser-Meyer-Olkin coefficient (KMO) and Bartlett’s test of sphericity. The EFA was conducted on the polychoric correlation matrix, using weighted least squares estimation and applying an oblimin rotation. To identify the appropriate number of factors to retain, parallel analysis and the empirical Kaiser criterion (EKC; Braeken and van Assen, 2017) were applied, in line with the recommendations of Auerswald and Moshagen (2019) for determining factor retention in exploratory factor analysis. Based on the EFA outcomes, items with primary factor loadings below 0.40 would be excluded from the analysis (Lloret-Segura et al., 2014).

After completing the exploratory phase of the questionnaire, the second subsample (*n*_2_ = 431) was employed to evaluate its internal structure by testing four alternative models: a four-factor ICM-CFA with items loading on single factors and no cross-loadings; a bifactor CFA where items loaded on both a general factor and one specific factor, with factors uncorrelated; an ESEM model using oblique rotation to allow small cross-loadings and more flexible estimation; and a bifactor-ESEM model combining a general and four specific factors, permitting cross-loadings among specific factors while constraining their correlations to zero. The ESEM approach enables more flexible and realistic modeling constraints, resulting in less biased factor loadings and inter-factor correlations (Asparouhov and Muthén, 2009), while the value of the bifactor model lies in its ability to determine unidimensionality in the presence of multidimensionality (Reise, 2012). All models were calculated using the diagonal weighted least squares with mean-and-variance adjusted chi-squared statistic (also known as WLSMV) (Satorra and Bentler, 1994), which is appropriate for analysing ordered categorical data (Maydeu-Olivares, 2001; Rhemtulla et al., 2012).

Model fit was evaluated following the criteria outlined by Brown (2015) and Kline (2023, 2024). Specifically, global model fit was assessed using the categorical maximum likelihood-estimated comparative fit index (CFI_cML_), Tucker-Lewis index (TLI_cML_), and root-mean-square error of approximation (RMSEA_cML_), as proposed by Savalei (2021). Additionally, the population-unbiased standardized root-mean-square residual (SRMR_u_) was calculated for each model (Shi et al., 2019). For the CFI_cML_ and TLI_cML_, values above 0.95 indicate excellent fit, while values above 0.90 suggest an acceptable fit. RMSEA_cML_ values below 0.08 reflect adequate fit, with values under 0.06 considered good. SRMR_u_ values below 0.08 were interpreted as indicating a good model fit.

Since the four proposed models were nested, model comparisons were conducted using the RMSEA_D_ statistic, as recommended by Savalei et al. (2024). Unlike the traditional change in RMSEA (ΔRMSEA), which is derived by subtracting the individual RMSEA values of the models, the RMSEA_D_ is computed based on the difference in their chi-square values. RMSEA_D_ can be interpreted similarly to a standard RMSEA, where lower values indicate smaller discrepancies in fit between the compared models (Savalei et al., 2024).

On the other hand, local model fit was assessed by examining absolute correlation residuals exceeding |0.10| for the same pair of variables, as such values may indicate potential model misspecifications (Maydeu-Olivares, 2017). Following Kline’s (2024) recommendations, the Benjamini-Hochberg (BH; Benjamini and Hochberg, 1995) procedure was applied to control multiplicity when performing significance testing of residuals. After adjusting *p*-values for multiple comparisons, only those residuals with significant values exceeding |0.10| were examined further in the final model.

Beyond evaluating fit indices, parameter estimates were also examined to identify the most suitable model among those tested. Following the guidelines of Morin et al. (2016a, 2016b), the initial step involved comparing the CFA and ESEM solutions by analysing factor loadings and factor correlations, favoring the model that showed lower correlations among the four dimensions (Swami et al., 2023). Subsequently, the chosen model was compared with its bifactor equivalent (either bifactor CFA or bifactor ESEM). Support for a bifactor model comes from the presence of a well-defined general (G) factor with strong loadings, along with reduced cross-loadings in the bifactor ESEM relative to the standard ESEM (Howard et al., 2016). Before this comparison, the strength and reliability of the general factor in the bifactor CFA and bifactor ESEM models were assessed using explained common variance (ECV), hierarchical omega (ωh), and the percentage of uncontaminated correlations (PUC)—the latter calculated only for the bifactor CFA model. Values exceeding 0.70 on these indices prove the existence of a solid general factor (Rodríguez et al., 2016).

Additionally, multigroup measurement invariance analyses were conducted on the full sample to assess the consistency of the factor structure across three sociodemographic groups: gender, job level, and educational level. Models testing configural invariance (equal factor structure), threshold invariance (equal thresholds), metric invariance (equal factor loadings), and strict invariance (equal residual variances) were estimated. For each step, it was determined whether imposing these constraints led to a significant decrease in model fit based on changes in the CFI, RMSEA, and SRMR indices (Chen, 2007): for threshold and metric invariance, |ΔCFI| < 0.010, |ΔRMSEA| < 0.015, |ΔSRMR| < 0.030; for strict invariance, |ΔCFI| < 0.010; |ΔRMSEA| < 0.015; |ΔSRMR| < 0.010. Nevertheless, since these cutoff values serve as guidelines rather than absolute rules (Putnick and Bornstein, 2016), the multigroup invariance analyses were complemented by reporting and interpreting the RMSEA_D_ values (Savalei et al., 2024).

Finally, the reliability of the dimensions of the JCQ questionnaire was evaluated using the composite reliability index, which has been proposed as a superior alternative to other measures (Rönkkö and Cho, 2022). In addition, the average extracted variance (AVE) was calculated in order to determine convergent validity. The acceptance criterion is that the average variance extracted of a construct must be greater than 0.50 (Hair et al., 2014), which means that the construct shares more than half of its variance with its indicators, and the rest of the variance is due to measurement error (Fornell and Larcker, 1981). All analyses were performed using Python code for meta-analyses, and for psychometric analyses, the packages “lavaan” (Rosseel, 2012), “semTools” (Jorgensen et al., 2022), and “psych” (Revelle, 2024) were used in R (version 4.4.2).

## 3 Results

### 3.1 Meta-analysis for reliability generalization

**Figure 2:**
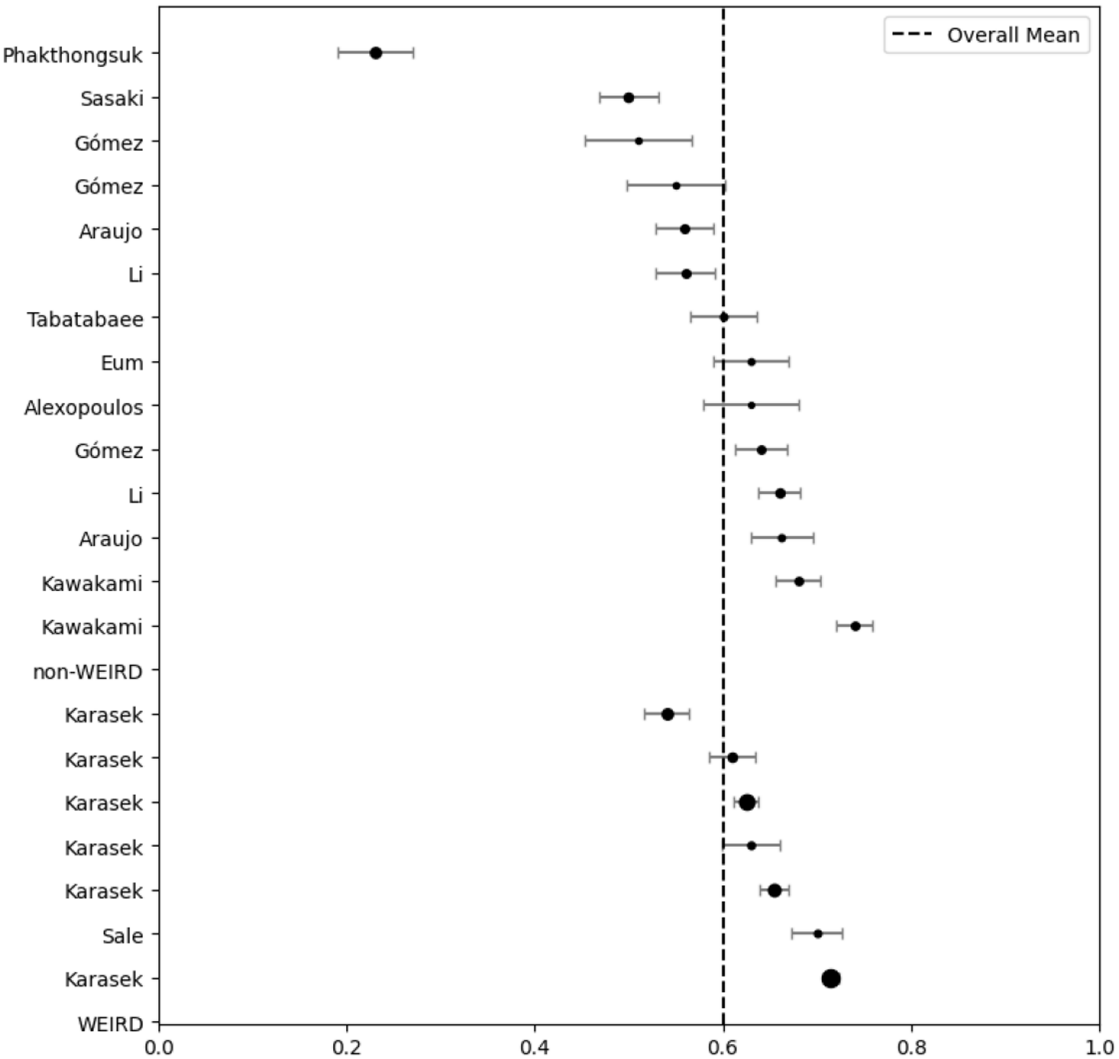
Psychological demands forest plot grouped by WEIRD status.

**Figure 3:**
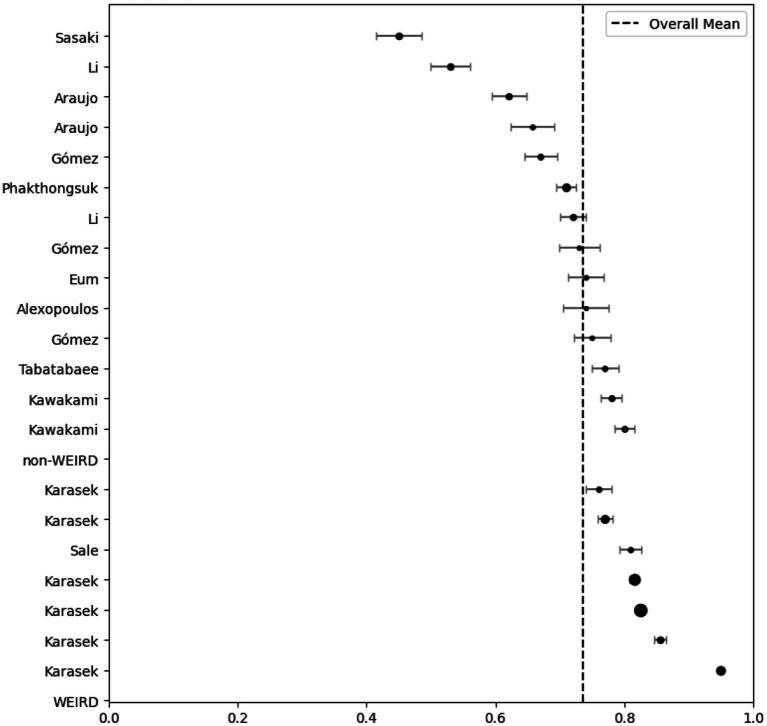
Job control forest plot grouped by WEIRD status.

**Figure 4:**
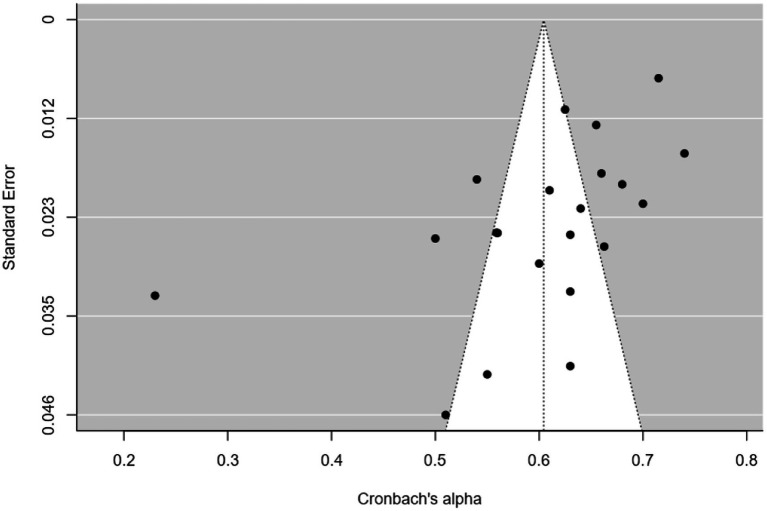
Psychological demands funnel plot.

**Figure 5:**
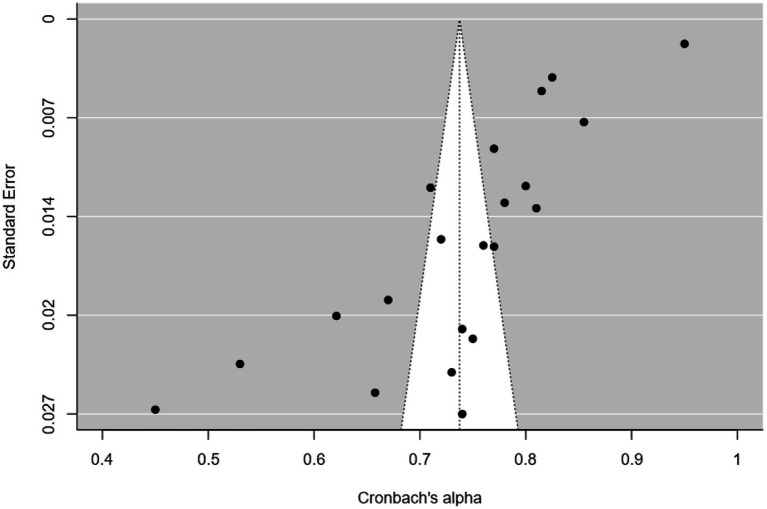
Job control funnel plot.

**Figure 6:**
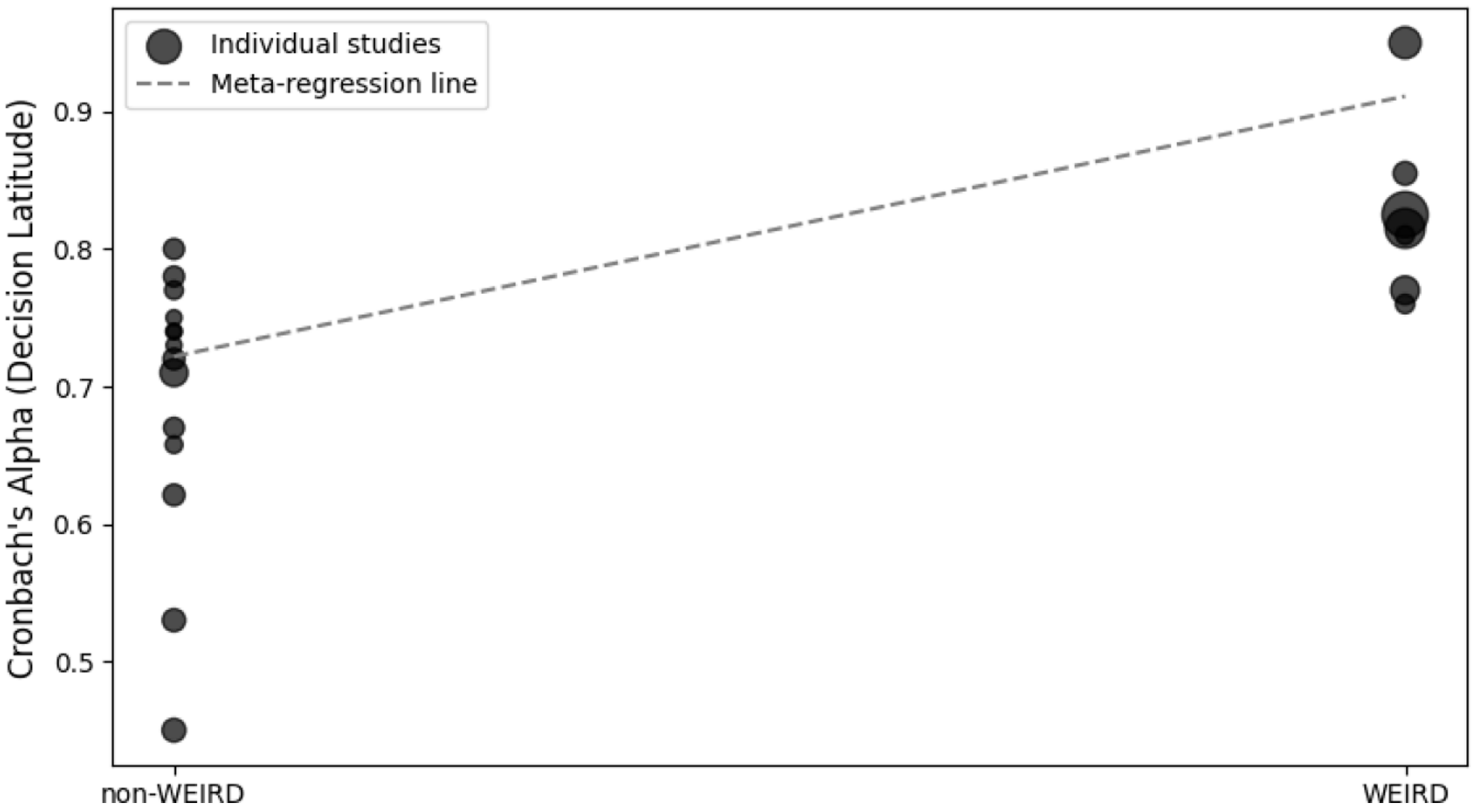
Meta-regression plot for job control moderated by WEIRD status.

Twenty-one studies (12 articles) evaluated the reliability of the dimensions of psychological demands (5 items) and job control (9 items) by Cronbach’s *α* (Table 1). The meta-analysis indicated that the psychological demands (*I^2^* = 97%; *Q =* 422.010; *p* < 0.001; *p* < 0.001; *N_Rosenthal_* = 159,504 > 115; *p* < 0.001) and job control (*I^2^* = 99%; *Q =* 3657.7; *p* < 0.001; *N_Rosenthal_* = 1,190,261 > 115; *p* < 0.001) dimensions had reliabilities of 0.603 (Figure 2) and 0.737 (Figure 3), respectively. Publication bias was also not detected (Figures 4, 5). The percentage of women was not a moderating variable for reliability. In contrast, regarding job control dimension, populations from WEIRD countries showed higher Cronbach’s α values than those from non-WEIRD countries, as shown in Figure 6 (*Moderator* = 0.183; *R^2^_Adj_* = 0.39; *p* < 0.001).

**Table 1:** Meta-analyzed reliability studies.

Author	Country	WEIRD status	*N* ^a^	Female (%)^b^	Psychological demands (Cronbach’s *α*)	Job control (Cronbach’s *α*)
1 Phakthongsuk and Apakupakul (2008)	Thailand	Non-WEIRD	1,415	48.9	0.230	0.710
2 Li et al. (2007)	China	Non-WEIRD	889	35.8	0.660	0.530
3 de Araújo and Karasek (2008)	Brazil	Non-WEIRD	780	49.1	0.559	0.621
4 de Araújo and Karasek (2008)	Brazil	Non-WEIRD	403	49.1	0.663	0.658
5 Sasaki et al. (2020)	Vietnam	Non-WEIRD	949	84.9	0.500	0.450
6 Alexopoulos et al. (2015)	Greece	Non-WEIRD	209	69.6	0.630	0.740
7 Gómez-Ortiz (2011)	Colombia	Non-WEIRD	294	100.0	0.550	0.750
8 Gómez-Ortiz (2011)	Colombia	Non-WEIRD	281	0.0	0.510	0.730
9 Gómez-Ortiz (2011)	Colombia	Non-WEIRD	661	54.2	0.640	0.670
10 Karasek et al. (1998)	USA	WEIRD	3,153	34.2	0.625	0.815
11 Karasek et al. (1998)	USA	WEIRD	4,298	39.3	0.715	0.825
12 Karasek et al. (1998)	Canada	WEIRD	949	42.6	0.610	0.855
13 Karasek et al. (1998)	Canada	WEIRD	1,946	48.8	0.655	0.950
14 Karasek et al. (1998)	Netherlands	WEIRD	1,506	29.9	0.540	0.770
15 Karasek et al. (1998)	Japan	Non-WEIRD	539	18.6	0.630	0.760
16 Li et al. (2004)	China	Non-WEIRD	774	75.1	0.560	0.720
17 Tabatabaee et al. (2013)	Iran	Non-WEIRD	490	73.1	0.600	0.770
18 Eum et al. (2007b)	Korea	Non-WEIRD	338	86.0	0.630	0.740
19 Kawakami and Fujigaki (1996)	Japan	Non-WEIRD	687	12.23	0.680	0.800
20 Kawakami and Fujigaki (1996)	Japan	Non-WEIRD	687	12.23	0.740	0.780
21 Sale and Kerr (2002)	Canada	WEIRD	484	89.0	0.700	0.810

a *N* = sample.

b Percentage of women in the sample.

### 3.2 Validation of the JCQ 15-item scale

**Figure 7:**
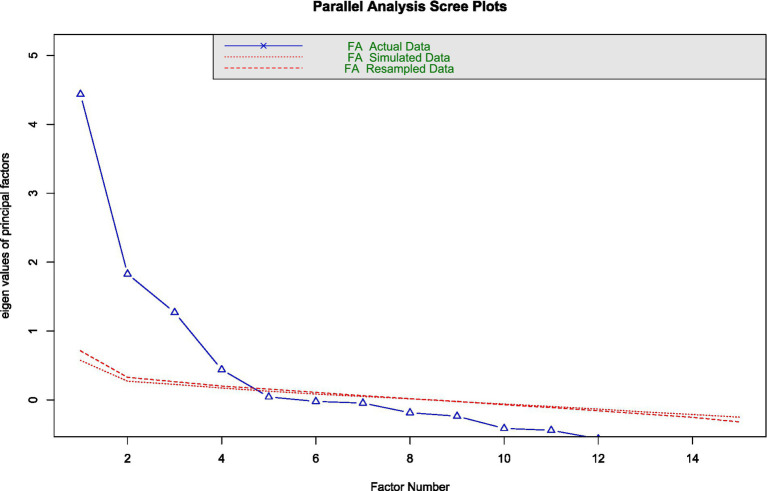
Parallel analysis results.

First of all, the chi-square test yielded no significant differences between the two random subsamples in terms of the distribution of variables such as gender (*χ*^2^ = 0.559, *p* = 0.455), job level (*χ*^2^ = 0.310, *p* = 0.857), educational level (*χ*^2^ = 5.825, *p* = 0.324), or employment sector (*χ*^2^ = 5.142, *p* = 0.399). This supports the fact that the random selection procedure successfully preserved equivalent distributions of sociodemographic characteristics across both groups. Therefore, an exploratory analysis of the questionnaire structure was performed with the first subsample (*n*_1_ = 429). The Kaiser-Meyer-Olkin (KMO) measure of sampling adequacy (0.81) and Bartlett’s test of sphericity (p < 0.001) indicated that the data were well-suited for exploratory factor analysis (EFA). In addition, both the parallel analysis (Figure 7) and the empirical Kaiser criterion (EKC) recommended a four-factor solution, which was subsequently used in the EFA.

The EFA results (Table 2) indicated that all items associated with a single factor had loadings ranging from 0.459 to 0.978. The highest observed cross-loading was |0.273|. Therefore, in line with the criteria suggested by Lloret-Segura et al. (2014), all items were retained for the subsequent CFA and ESEM analyses. Overall, the EFA supported a four-factor, 15-item structure which accounted for 66% of the total variance. The four extracted factors were also theoretically coherent based on the content of the corresponding items.

**Table 2:** Rotated factor loadings matrix.

Item	Mean (*SD*)	F1	F2	F3	F4
K1	3.083 (0.723)	−0.020	0.000	**0.859**	0.062
K2	3.436 (0.675)	−0.062	0.038	0.197	**0.671**
K3	2.565 (0.918)	0.054	**0.727**	0.142	0.034
K4	2.792 (0.817)	**0.796**	0.062	0.031	0.144
K5	3.503 (0.657)	0.027	0.058	−0.008	**0.816**
K6	3.109 (0.824)	−0.010	−0.011	**0.906**	−0.034
K7	2.392 (0.847)	−0.096	**0.525**	−0.007	0.017
K8	2.369 (0.769)	**0.958**	−0.093	−0.056	−0.023
K9	3.306 (0.706)	−0.036	−0.059	−0.039	**0.978**
K10	2.851 (0.885)	−0.056	**0.918**	−0.071	0.030
K11	2.723 (0.877)	**0.622**	0.273	0.096	−0.124
K12	3.114 (0.710)	0.100	0.088	0.005	**0.773**
K13	2.608 (0.814)	0.266	**0.560**	0.078	−0.024
K14	2.969 (0.790)	0.094	−0.046	0.033	**0.459**
K15	3.227 (0.734)	0.011	0.000	**0.885**	−0.003

*n*_1_ = 429; SD, standard deviation; F1, psychological demands; F2, skill discretion; F3, decision authority; F4, social support. EFA was calculated on the polychoric correlation matrix, using weighted least squares estimation and oblimin rotation. Factor loadings >0.40 are in bold.

To evaluate validity evidence based on internal structure, the four structural equation models were tested with the second subsample (*n*_2_ = 431) and compared using nested model fit analysis. None of the models exhibited Heywood cases, as there were no negative error variances or *R*^2^ values exceeding 1. The fit indices for the four tested models—four-factor CFA, four-factor ESEM, bifactor CFA, and bifactor ESEM—are presented in Table 3.

**Table 3:** Fit indices of the different models for the JCQ questionnaire and comparison of nested models.

Tested model	*df*	Global fit					Local fit	RMSEA_D_ (90% CI)
		*χ* ^2^	RMSEA_cML_ (90% CI)	CFI_cML_	TLI_cML_	SRMR_u_	CR	
Four-factor CFA	84	381.493	0.113 (0.099, 0.127)	0.875	0.844	0.071	19.05%	
Bifactor CFA	75	311.129	0.092 (0.076, 0.108)	0.926	0.900	0.066	16.19%	0.093 (0.066, 0.122)
Four-factor ESEM	51	78.288	0.063 (0.038, 0.087)	0.976	0.951	0.014	0%	0.141 (0.125, 0.158)
Bifactor ESEM	40	37.650	0.018 (0.000, 0.061)	0.998	0.996	0.006	0%	0.024 (0.0.00, 0.059)

*n*_2_, 431; df, degrees of freedom; *χ*^2^, scaled chi-square; RMSEA_cML_, root mean square error of approximation; CFI_cML_, comparative fit index; TLI_cML_, Tucker-Lewis index; SRMR_u_, standardized root mean square residual; CR, percentage of significant absolute correlation residuals greater than |0.10|; RMSEA_D_, root mean square error of approximation associated with the *χ*^2^ difference test, each index is obtained from comparison with the previous model in the table. The *p*-values associated with the *χ*^2^ test of fit are below 0.05 in all models except for the bifactor ESEM model.

As shown, the four-factor CFA model presented inadequate global fit indices. In contrast, the bifactor CFA, four-factor ESEM, and bifactor ESEM models showed better global fit indices, which was particularly reflected in higher CFI and TLI values. A more detailed examination of the differences in model fit using the RMSEA_D_ index confirmed that the four-factor CFA model had the worst fit, with an RMSEA_D_ of 0.093 compared to the bifactor CFA model. Furthermore, the RMSEA_D_ obtained between the four-factor ESEM model and the bifactor CFA model was 0.141, indicating a better fit for the ESEM solution. Finally, the bifactor ESEM model showed a fit comparable to the four-factor ESEM, with a RMSEA_D_ of 0.024 between the two models.

**Figure 8:**
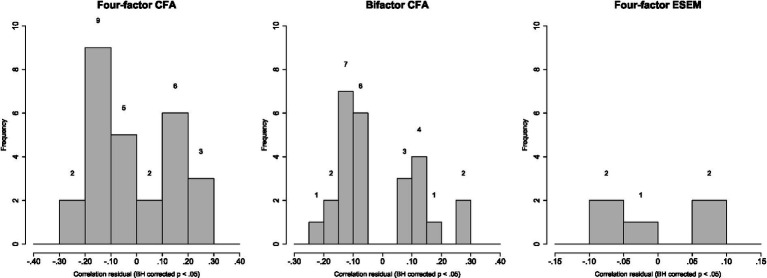
Histogram for correlation residuals significant at BH corrected *p* < 0.05 for the four-factor CFA model, bifactor CFA model and four-factor ESEM model.

However, the choice of a model should not rely solely on global fit indices, as these reflect the central tendency of residuals, indicating where most values are located, and not capturing their variability or dispersion. Therefore, evaluating the local fit of the models is equally important, which can be done by analysing correlation residuals (Kline, 2024). In the case of the four models assessed, with 15 observed variables (items), a total of 105 correlation residuals were examined. After applying the Benjamini-Hochberg (BH) procedure to control for multiple comparisons in the significance testing of residuals, results shown in Figure 8 indicate that both the four-factor CFA and bifactor CFA models exhibited a greater number of significant correlation residuals exceeding |0.10|. On the other hand, the four-factor ESEM model did not obtain significant correlation residuals exceeding the value |0.10|, while the bifactor ESEM model did not present significant correlation residuals after applying the BH correction (for this reason, it has been omitted from the graphical representation in Figure 8).

More specifically, the four-factor CFA model yielded 27 significant correlation residuals, with 20 exceeding |0.10| (representing 19.05% of the total correlation residuals). Similarly, the bifactor CFA model showed 26 significant correlation residuals, of which 17 surpassed the absolute value of |0.10| (16.19%). In contrast, the four-factor ESEM model had only 5 significant correlation residuals, none exceeding |0.10|. In summary, these findings indicate that the four-factor ESEM and bifactor ESEM models demonstrated superior local fit compared to the four-factor CFA and bifactor CFA models.

In the final step of model evaluation, and in line with the recommendations by Morin et al. (2016a, 2016b), first the parameter estimates of the four-factor CFA and four-factor ESEM models were compared. Table 4 presents the factor loadings, cross-loadings, and uniquenesses for each item in both models, while Table 5 displays the factor correlations. As shown in Table 4, the magnitude of the target factor loadings was similar across the two models, with the four-factor CFA showing loadings ranging from 0.414 to 0.909 (*M* = 0.767), and those of the four-factor ESEM ranging from 0.487 to 0.970 (*M* = 0.762), indicating well-defined latent dimensions in both cases. In the ESEM model, target loadings were consistently higher than cross-loadings, which were generally minimal (|*λ*| = 0.002 to 0.279; |*M*| = 0.069). Notably, only three cross-loading exceeded |0.20|: item 13 from the factor skill discretion showed a cross-loading of 0.248 on the psychological demands factor, compared to a standardized loading of 0.604 on its intended dimension; item 11 from the factor psychological demands showed a cross-loading of 0.279 on the skill discretion factor, compared to a standardized loading of 0.641 on its intended dimension; item 2 from the factor social support showed a cross-loading of 0.226 on the decision authority factor, compared to a standardized loading of 0.536 on its intended dimension. Upon qualitative review of the content of items 2, 11, and 13, these cross-loadings on the aforementioned factors appear reasonable. All remaining cross-loadings were below |0.141|.

**Table 4:** Standardized factor loadings (λ) and uniquenesses (δ) for the four-factor CFA and four-factor ESEM.

Items	Four-factor CFA		Four-factor ESEM				
	λ	δ	Factor 1 (*λ*)	Factor 2 (λ)	Factor 3 (λ)	Factor 4 (λ)	δ
1. Psychological demands							
K4	0.904	0.183	**0.861**	−0.057	0.052	0.135	0.241
K8	0.757	0.427	**0.918**	−0.100	−0.086	−0.038	0.204
K11	0.800	0.360	**0.641**	0.279	0.096	−0.125	0.393
2. Skill discretion							
K3	0.785	0.383	−0.060	**0.700**	0.135	0.040	0.431
K7	0.414	0.829	−0.030	**0.587**	−0.089	0.035	0.449
K10	0.770	0.406	−0.057	**0.896**	−0.053	0.010	0.243
K13	0.754	0.431	0.248	**0.604**	0.007	0.037	0.468
3. Decision authority							
K1	0.909	0.174	−0.029	0.040	**0.822**	0.099	0.227
K6	0.876	0.233	−0.012	0.035	**0.908**	−0.029	0.180
K15	0.819	0.330	0.084	−0.051	**0.827**	0.018	0.304
4. Social support							
K2	0.732	0.465	−0.031	−0.019	0.226	**0.536**	0.474
K5	0.777	0.397	0.135	−0.030	−0.079	**0.836**	0.341
K9	0.891	0.207	−0.045	−0.026	−0.057	**0.970**	0.119
K12	0.898	0.193	−0.002	0.141	−0.008	**0.827**	0.225
K14	0.421	0.823	−0.106	−0.005	−0.051	**0.491**	0.771

*n*_2_ = 431. Boldface indicates target ESEM factor loadings.

**Table 5:** Standardized factor correlations for the four-factor CFA and four-factor ESEM.

Dimensions	1	2	3	4
1. Psychological demands		0.415^**^	0.196^**^	0.081
2. Skill discretion	0.283^**^		0.370^**^	0.408^**^
3. Decision authority	0.130^*^	0.311^**^		0.496^**^
4. Social support	0.046	0.334^**^	0.420^**^	

*n*_2_ = 431. Four-factor CFA correlations are shown above the diagonal; four-factor exploratory structural equation modeling correlations are shown below the diagonal. *p < 0.05; **p < 0.01.

The correlations between factors (see Table 5) were slightly lower in the four-factor ESEM model (*r* = 0.046 to 0.334; |*M*| = 0.254) compared to the four-factor CFA model (*r* = 0.081 to 0.496; |*M*| = 0.328), which supports the suitability of the ESEM solution (Swami et al., 2023). Importantly, the overall pattern of factor correlations remained consistent across both models. Two key findings emerged from this factor correlation analysis: first, the association between psychological demands and social support was very weak and non-significant in both models; second, although statistically significant, the correlation between skill discretion and decision authority was modest in magnitude. Notably, the strongest factor correlations were observed between decision authority and social support. Taking into account the superior global and local fit of the four-factor ESEM model and the lower factor correlations, the four-factor ESEM solution was retained for subsequent comparison with the bifactor ESEM model.

Regarding the bifactor models, Table 6 shows the parameter estimates for both the bifactor CFA and bifactor ESEM solutions. Although both models demonstrated acceptable global fit indices—with the bifactor ESEM showing superior values—it is important to exercise caution, as bifactor models have a known tendency to overfit the data regardless of whether the population model has a bifactor structure or not (Bonifay and Cai, 2017; Bonifay et al., 2017; Markon, 2019). Therefore, model evaluation should not rely solely on global fit measures. In this regard, Table 6 reveals that the general factor in both bifactor solutions is not strongly represented, as several items exhibited loadings below 0.30 on the general factor (Swami et al., 2023). This observation is further supported by the low explained common variance (ECV) and hierarchical omega (ωh) values. Although the PUC value for the bifactor CFA model was 0.790 (exceeding the proposed threshold of 0.70), the ECV values for the bifactor CFA and bifactor ESEM were only 0.355 and 0.326, respectively, falling short of the 0.70 cutoff required to confirm a well-defined general factor (Rodríguez et al., 2016). Similarly, the values of the ωh of the bifactor CFA (ωh = 0.579) and bifactor ESEM (ωh = 0.576) were below the cutoff point of 0.70 to support the existence of a strong general factor. In line with these results, the bifactor ESEM model did not reduce cross-loadings (|*λ*| = 0.002 to 0.360; |*M*| = 0.072) but slightly increased them with respect to the ESEM solution (|*λ*| = 0.002 to 0.279; |*M*| = 0.069).

**Table 6:** Standardized factor loadings (λ) and uniquenesses (δ) for bifactor CFA and bifactor ESEM.

Items	Bifactor CFA			Bifactor ESEM					
	G-Factor (λ)	S-Factor (λ)	δ	G-Factor (λ)	S-Factor 1 (λ)	S-Factor 2 (λ)	S-Factor 3 (λ)	S-Factor 4 (λ)	δ
1. Psychological demands									
K4	0.349	0.780	0.269	0.383	**0.836**	−0.044	−0.088	−0.019	0.144
K8	0.036	0.936	0.123	0.074	**0.843**	−0.005	−0.080	−0.090	0.269
K11	0.397	0.613	0.467	0.124	**0.717**	0.360	0.218	−0.009	0.293
2. Skill discretion									
K3	0.553	0.521	0.423	0.619	−0.020	**0.562**	−0.083	−0.083	0.286
K7	0.208	0.438	0.765	0.203	0.023	**0.412**	−0.078	0.039	0.781
K10	0.467	0.730	0.250	0.343	0.059	**0.778**	0.018	0.089	0.265
K13	0.517	0.443	0.536	0.334	0.311	**0.557**	0.047	0.075	0.474
3. Decision authority									
K1	0.671	0.554	0.243	0.767	−0.063	−0.056	**0.484**	−0.029	0.169
K6	0.575	0.711	0.164	0.600	−0.011	0.013	**0.667**	−0.007	0.195
K15	0.543	0.633	0.305	0.510	0.079	−0.030	**0.681**	0.057	0.265
4. Social support									
K2	0.619	0.359	0.487	0.642	−0.081	−0.080	0.123	**0.346**	0.441
K5	0.456	0.645	0.376	0.474	0.087	−0.005	−0.061	**0.652**	0.339
K9	0.452	0.838	0.093	0.500	−0.087	0.004	−0.002	**0.786**	0.125
K12	0.592	0.650	0.227	0.503	−0.015	0.167	0.073	**0.713**	0.205
K14	0.180	0.436	0.778	0.292	−0.137	−0.032	−0.093	**0.353**	0.762

*n*_2_ = 431; G-Factor = general factor; S-Factor = specific factors. Boldface indicates bifactor ESEM target S-factor loadings.

In conclusion, the results obtained thus far support the selection of the four-factor ESEM model as the most appropriate representation of the data. Compared to the four-factor CFA model, the ESEM solution demonstrated significantly better global fit indices, improved local fit, and lower factor correlations. Although the bifactor ESEM model achieved the best global and local fit among all models, it failed—like the bifactor CFA—to demonstrate a clearly defined general factor. Furthermore, the bifactor ESEM model slightly increased item cross-loadings on the specific factors rather than reducing them, which further undermines its theoretical advantage. Therefore, considering both empirical performance and theoretical coherence, the four-factor ESEM model emerges as the most appropriate factor solution.

To evaluate the stability of the factor structure, multigroup invariance analyses were conducted using three sociodemographic variables. Specifically, these analyses examined measurement invariance across gender (male and female), job level, recoded into two approximately equal-sized groups (basic workers and managers/supervisors), and educational level, recoded into three groups of similar size (secondary education, vocational training, and university studies). As a preliminary step, Table 7 presents the global fit indices for the four-factor ESEM model estimated separately within each of these subgroups.

**Table 7:** Fit indices of the four-factor ESEM models for each of the subsamples generated by the sociodemographic variables used in the multigroup measurement invariance analysis.

Group	*n*	*χ* ^2^	RMSEA_cML_ (90% CI)	CFI_cML_	TLI_cML_	SRMR_u_
Women	409	107.924	0.053 (0.039, 0.066)	0.975	0.949	0.016
Men	451	140.841	0.063 (0.051, 0.076)	0.965	0.928	0.017
Basic level workers	461	119.311	0.055 (0.042, 0.068)	0.973	0.944	0.018
Managers/supervisors	399	154.781	0.072 (0.059, 0.085)	0.955	0.908	0.021
Secondary education	309	145.203	0.077 (0.062, 0.091)	0.950	0.900	0.021
Vocational training	282	99.096	0.058 (0.041, 0.075)	0.967	0.932	0.019
University studies	269	104.824	0.064 (0.047, 0.082)	0.965	0.930	0.017

*n* = number of subjects per subsample; *χ*^2^, scaled chi-square; RMSEA_cML_, root mean square error of approximation; CFI_cML_, comparative fit index; TLI_cML_, Tucker-Lewis index; SRMR_u_, standardized root mean square residual. The *p*-values associated with the *χ*^2^ test of fit are 0.000 in all four models.

To conduct the multigroup measurement invariance analysis, the sequential steps outlined by Kline (2023) were followed, beginning with configural invariance (equal factor structure), followed by threshold invariance (equal item thresholds), metric invariance (equal factor loadings), and finally, strict invariance (equal residuals). It is important to note that when total invariance is not assumed (a common situation in social science research), it is possible to explore partial invariance of models by relaxing the restrictions on one or more parameters. Therefore, since the operationalization of the ESEM precludes its use in partial invariance estimation, the ESEM-within-CFA approach (EWC; Marsh et al., 2013) was used to perform the invariance analyses, with the aim of allowing the calculation of partial invariance if necessary. The EWC basically consists of transforming the ESEM solution into the standard CFA framework in order to perform the analyses mentioned above (Morin et al., 2013).

Table 8 shows the results of the multigroup measurement invariance analysis performed with the sociodemographic grouping variables. As can be seen, the configurational models obtained satisfactory fit indices, which supports the presence of configurational invariance across groups. Regarding threshold invariance, the differences between the RMSEA, CFI and SRMR indices in the three multigroup analyses were smaller than the criterion determined by Chen (2007), while the RMSEA_D_ values obtained between the configurational and threshold models also support the presence of metric invariance, both for the multigroup analysis by gender (RMSEA_D_ = 0.074), for the multigroup analysis by job level (RMSEA_D_ = 0.061) and for the multigroup analysis by educational level (RMSEA_D_ = 0.066). Regarding metric invariance, the changes in the RMSEA, CFI and SRMR indices were small and did not reach the limit considered to rule out metric invariance, while the RMSEA_D_ values were less than 0.080 in the multigroup analysis by gender (RMSEA_D_ = 0.062), in the multigroup analysis by job level (RMSEA_D_ = 0.071) and in the multigroup analysis by educational level (RMSEA_D_ = 0.035). Finally, with respect to strict invariance, the results presented in Table 8 show that this type of invariance is clearly met for the multigroup analysis by gender and job level. In this sense, the changes produced in the RMSEA, CFI and SRMR fit indices between the metric and strict models were not large enough to rule out the presence of strict invariance in the comparisons by gender or job level. Furthermore, paying attention to the value of the RMSEA_D_ statistic, this index also supported the presence of strict invariance in the multigroup analyses by gender (RMSEA_D_ = 0.054) and in the multigroup analyses by job level (RMSEA_D_ = 0.059). However, strict invariance by educational level was not so clear from a mere inspection of the results. In this case, although the changes in the RMSEA and SRMR indices did not exceed the threshold established for rejecting this type of invariance, the difference between the CFI values of the metric and strict models was 0.014, which is higher than the cutoff point of 0.010 proposed by Chen (2007). However, inspection of the RMSEA_D_ index also supports the presence of strict invariance by educational level (RMSEA_D_ = 0.063), so it is concluded that this level of invariance can also be maintained.

**Table 8:** Multigroup measurement invariance analysis results.

Levels of invariance	*χ* ^2^	RMSEA_cML_ (90% CI)	CFI_cML_	TLI_cML_	SRMR_u_	RMSEA_D_ (90% CI)
Gender						
Configurational	248.877	0.058 (0.049, 0.067)	0.970	0.938	0.018	
Threshold	285.568	0.060 (0.051, 0.068)	0.964	0.935	0.023	0.074 (0.048, 0.100)
Metric	371.331	0.060 (0.052, 0.068)	0.954	0.934	0.042	0.062 (0.046, 0.078)
Strict	437.684	0.059 (0.052, 0.066)	0.947	0.935	0.046	0.054 (0.035, 0.072)
Job level						
Configurational	273.782	0.063 (0.054, 0.072)	0.964	0.926	0.024	
Threshold	303.187	0.063 (0.054, 0.071)	0.955	0.923	0.025	0.061 (0.034, 0.089)
Metric	404.279	0.064 (0.057, 0.072)	0.946	0.923	0.048	0.071 (0.056, 0.086)
Strict	476.914	0.064 (0.057, 0.071)	0.938	0.924	0.049	0.059 (0.041, 0.077)
Educational level						
Configurational	348.095	0.067 (0.058, 0.076)	0.959	0.917	0.025	
Threshold	401.688	0.066 (0.055, 0.074)	0.953	0.918	0.028	0.066 (0.041, 0.090)
Metric	481.127	0.059 (0.051, 0.067)	0.950	0.935	0.043	0.035 (0.012, 0.052)
Strict	608.288	0.061 (0.054, 0.068)	0.936	0.931	0.044	0.063 (0.047, 0.079)

*N* = 860; *χ*^2^, scaled chi-square; RMSEA_cML_, root mean square error of approximation; CFI_cML_, comparative fit index; TLI_cML_, Tucker-Lewis index; SRMR_u_, standardized root mean square residual; RMSEA_D_, root mean square error of approximation associated with the *χ*^2^ difference test, each index is obtained from comparison with the previous model in the table. The *p*-values associated with the *χ*^2^ test of fit are 0.000 for all models.

Finally, the reliability and convergent validity analyses performed on the dimensions of the JCQ scale in this study are presented and discussed. As can be seen in Table 9, the dimensions of the scale obtained very good composite reliability indices, all of them between 0.80 and 0.89. In addition, the average variance extracted (AVE) values for all four dimensions exceeded the recommended threshold of 0.50, meaning that each construct explains more than half of the variance of its indicators on average, which supports the adequate convergent validity of the scale.

**Table 9:** Composite reliability index and average variance extracted of the four dimensions.

Dimensions	Composite reliability (CR)	Average variance extracted (AVE)
1. Psychological demands	0.853	0.665
2. Skill discretion	0.800	0.510
3. Decision authority	0.890	0.728
4. Social support	0.862	0.570

*N* = 860.

## 4 Discussion

The findings of this study substantiate the revised theoretical framework (Fransson et al., 2012; Karasek et al., 1998; Karasek, 1979; Theorell, 1996, 2014; Theorell et al., 1988). Although the results of the meta-regression indicate that the effect of phenotypic sex (male or female) is non-existent when evaluating both dimensions, more relevant is the finding that confirms that the reliability of the job control dimension varies when taking into account whether or not the population belongs to a WEIRD country. However, this finding will be examined further as the understanding of each dimension of the JCQ is expanded in the following sections.

Regarding the psychometric study, the validation process followed has made it possible to obtain a 15-item scale with appropriate fit indices. First, by parallel analysis and the EKC, the number of factors to be retained in the EFA was determined. As a result, it could be found that the four-factor structure supported by these analyses coincided with the structure proposed by other researchers (Sanne et al., 2005). On the other hand, it should be noted that the social support dimension has not been divided into the subfactors of support from supervisors and support from colleagues, as has been the case in other studies (Alexopoulos et al., 2015). That is, the four-factor structure of the JCQ validated in this study allows us to represent the population analysed, considering the differentiation of the job control dimension into the subdimensions of skill discretion and decision authority.

On the other hand, to evaluate the internal structure of the scale, the four-factor CFA, bifactor CFA, four-factor ESEM and bifactor ESEM models were tested. The results obtained show positive fit indices in all models analysed, except for the four-factor CFA model. Thus, the fit indices corresponding to the four-factor ESEM model and the bifactor ESEM model are significantly higher than those of the four-factor CFA and bifactor CFA models. However, a more in-depth analysis of the bifactor ESEM model did not provide sufficient support to guarantee the existence of a general factor, so the four-factor ESEM model was ultimately retained as the one that best represented the structure of 15-item JCQ.

At the same time, and in line with the above, the multigroup measurement invariance analyses support the non-existence of significant differences in the interpretation of the scale according to gender, job level or educational level, which evidences the stability of the model and facilitates the utilization of the questionnaire as a useful measurement tool. It is important to note, however, that although measurement invariance was achieved in this study, this does not guarantee that the same will occur in every context. As recent literature highlights, strict thresholds for invariance are often difficult to achieve, and partial or non-invariance can offer valuable theoretical and cultural insights rather than being seen solely as a limitation (Kusano et al., 2025; Sterner et al., 2024). Therefore, the invariance analyses presented here should be considered an indispensable step in this study’s validation process, but future applications of the scale in new populations should continue to test for invariance to ensure its proper use and contextual relevance.

The first dimension of the scale corresponds completely to that defined by Karasek et al. (1998) and also used by Fransson et al. (2012): psychological demands. This, together with five-item version of the meta-analysis that evaluated the reliability of the systematically reviewed primary research studies, allows to state that psychological demands are defined as the degree to which a job can be stressful or emotionally exhausting. It includes items such as “working too fast” or “working too intensely.” The key concept here would be “frantic.” That is, the interaction between the number of tasks and the time needed to complete them. If the job requires attending to many tasks in a short time, it would be frantic. This conceptualization of psychological demands as a robust and clearly defined factor is consistent with findings from a wide range of cultural contexts. For example, validation studies of the JCQ in Asian countries such as China (Li et al., 2004) and Iran (Tabatabaee et al., 2013) have also reported good reliability for this dimension, suggesting that the experience of work intensity and time pressure is a relatively universal construct across different work cultures. Similarly, research in European contexts also corroborates the stability of this five-item scale (Formazin et al., 2025).

In this line, the second dimension obtained corresponds to the subdimension of skill discretion, or the variety of habilities and competences that can be used in the job (Karasek et al., 1998). This worker’s control over the performance of his or her own job is achieved when new things are learned and when the work is not repetitive. The third dimension aligns with decision authority: the autonomy that the worker has to decide what to do and how to do it (Karasek et al., 1998). In this case, the worker’s control over the performance on his or her own job is achieved when he or she is free to decide what to do, how much to do and how to do it. It should be noted that version B of the JCQ validated by Fransson et al. (2012) only assesses the dimension of the ability to decide how to do the job. The differentiation of job control into these two subdimensions is a critical finding of the present study, and it aligns with research conducted in other diverse national contexts. For instance, studies in other European countries have also supported a two-factor structure for job control (Sanne et al., 2005), as has research in Latin American contexts (Gómez-Ortiz, 2011). This suggests that the distinction between having the autonomy to make decisions (decision authority) and the opportunity to use one’s skills (skill discretion) is a meaningful one across various cultures. However, it is worth noting that this two-factor structure is not universally found; some studies, particularly in certain Asian contexts (Li et al., 2007; Phakthongsuk and Apakupakul, 2008; Sasaki et al., 2020), have reported a better fit for a unidimensional job control factor, possibly reflecting cultural differences in organizational hierarchies and job design.

Finally, the fourth dimension identified in the scale corresponds to social support, a construct that was later incorporated into the original Job Demand-Control model (Johnson and Hall, 1988; Johnson et al., 1989). Social support refers to the degree to which workers perceive that they receive emotional and instrumental assistance from supervisors and colleagues. This dimension plays a protective role in buffering the negative effects of high job demands and low control, acting as a key moderating factor in the development of job strain (Häusser et al., 2010). The inclusion of this factor in this 15-item version of the JCQ reinforces the importance of considering relational aspects of the work environment when evaluating psychosocial risks. Recent research continues to underscore that social support at work is not only critical for reducing stress and burnout, but also positively associated with job satisfaction, engagement, and organizational commitment (Kossek et al., 2011; Rattrie et al., 2020). Its presence in the final factorial solution thus highlights the multidimensional nature of work stress and the necessity of including interpersonal resources in the assessment of job quality.

About populations from WEIRD countries, results showed higher Cronbach’s *α* values than those from non-WEIRD countries for the job control dimension. This disparity is likely attributed to fundamental cultural differences impacting the conceptualization and experience of autonomy and control in the workplace. In WEIRD cultures, characterized by lower power distance and higher individualism, the constructs of skill discretion and decision authority are more aligned with societal values and prevalent work structures, leading to a more consistent interpretation and reporting of job control items (House et al., 2004). Consequently, the internal consistency of the scale (as measured by Cronbach’s α) is higher. Conversely, in many non-WEIRD contexts, higher power distance and collectivism may render the concept of individual job control less salient or even incongruent with hierarchical organizational norms, leading to more varied interpretations and thus lower internal reliability of the scale. This effect is not observed for the psychological demand dimension, which appears to be a more universally understood and experienced aspect of work intensity and workload, transcending specific cultural values related to autonomy (Karasek and Theorell, 1990).

In summary, the meta-analyses and validation presented in this research reflect a collaborative effort between WEIRD and non-WEIRD countries. It is a contribution to the decentralization of psychometric production. As noted by Beyebach et al. (2021), this addresses the lack of stable research networks and the high author turnover in the field. In line with the promotion of psychometric assessment in non-WEIRD countries, this research emphasizes that validity is not inherent to a test, but contextual. It also demonstrates that rigorous instrument development is possible from underrepresented regions. Notably, our meta-analysis found no studies using Spanish samples. A 56% of the included studies came from non-WEIRD countries, suggesting a potential reversal of the typical trend in this domain.

These collaborative efforts enhance psychometric robustness—clarifying which JCQ dimensions are stable across cultures and which require adaptation—while promoting a more inclusive science. Co-producing knowledge with local researchers supports cultural equivalence and shared intellectual authorship, aligning with calls for a more global and equitable psychology (Anjum and Aziz, 2024; Medin and Bang, 2014). An example of this is Gómez-Ortiz’s (2011) validation in Colombia, based on Escribà-Agüir et al. (2001) work with Spanish nurses. The latter was not included in our meta-analysis due to the use of an extended version of JCQ.

As limitations and suggestions, we can mention the following. First, although the total sample size is considerable and allows researchers to obtain relevant conclusions, the fact that these results were obtained with a sample of working people belonging to the Spanish labor context may make the generalization of these data require caution, since the labor situation present in the Spanish society may be very different from the labor realities of other countries. In another vein, although this research has demonstrated the invariance of the questionnaire through three sociodemographic factors, it would also be convenient to perform multigroup measurement invariance analysis to examine the stability of the factor structure across some other demographic variables (for instance, with different sectors or with different working conditions, such as the type of employment contract). This was not possible in this study because generating more groupings based on sociodemographic characteristics would have provided very unequal groups, which could have biased the results. Therefore, another possible line of research of great relevance corresponds to the analysis of the performance of this scale with workers from different sectors and types of employment contract, to verify whether this invariance is indeed maintained or whether there are substantial differences. Finally, the study’s cross-sectional nature limits the possibility of examining the stability of the instrument over time. Future research could address this by employing a longitudinal design to assess test–retest reliability. Also, the use of self-report instruments may introduce potential biases—such as recall inaccuracies or the influence of social desirability—which could affect the reliability of responses. Future research is encouraged to incorporate complementary measures designed to detect and control for these sources of bias.

## 5 Conclusion

More than a decade has passed since Fransson et al. (2012) conducted their comparative work on different versions of the psychological demands and job control scales, based on the Job Content Questionnaire (JCQ) and the Demand Control Questionnaire (DCQ). Importantly, this study also contributes to the literature by providing a comprehensive meta-analysis of the reliability and performance of the JCQ dimensions across a wide range of international samples (Part I). This synthesis not only updates and expands previous comparative work (e.g., Fransson et al., 2012) but also highlights critical findings, such as the cultural stability of the psychological demands dimension and the variability of job control between WEIRD and non-WEIRD contexts. These results offer valuable insight into how these constructs are experienced across different labor markets. Building on these findings, this paper provides results on a version with a generalizable validity and reliability of these scales. In addition, this research confirms the psychometric properties of an abbreviated version of the JCQ, establishing it as a useful and reliable instrument for evaluating essential psychosocial dimensions of work, such as psychological demands, decision authority, skill discretion and social support. Beyond its scientific contribution, the tool offers practical value for workplace assessment and intervention. Managers and occupational health professionals may find it especially helpful for identifying psychosocial risks, monitoring work environments, and designing strategies to promote employee well-being and prevent stress-related outcomes. Given its brevity and focus on core job characteristics, the scale is also suitable for use in diverse organizational settings, particularly in sectors marked by high emotional or cognitive demands—such as healthcare, education, or frontline service industries—where job conditions can directly impact staff health and well-being, motivation and retention.

## Data Availability

The data analyzed in this study is subject to the following licenses/restrictions: The raw data supporting the conclusions of this article will be made available by the authors, without undue reservation. Requests to access these datasets should be directed to adrian.garcias@umh.es.
