# Supplementary material for: Psychosocial work environment beyond WEIRD: meta-analytic and psychometric evidence on the Job Content Questionnaire
Source: Front Psychol. 2025 Oct 2;16:1642607. doi: 10.3389/fpsyg.2025.1642607 (PMC12528039; doi:10.3389/fpsyg.2025.1642607)
Supplement: Supplementary file 1 [file Data_Sheet_1.PDF]

Marque su respuesta con una cruz (X), utilizando la escala que acompaña a cada afirmación.

**1**<br>Totalmente en desacuerdo	**2**<br>Moderadamente en desacuerdo	**3**<br>Moderadamente de acuerdo	**4**<br>Totalmente de acuerdo

K1.	Tengo libertad para decidir lo que hago en mi trabajo	1	2	3	4
K2.	Tengo buenas relaciones con mi jefe/supervisor(es)	1	2	3	4
K3.	Mi trabajo requiere que sea creativo	1	2	3	4
K4.	El ritmo de mi trabajo es muy intenso	1	2	3	4
K5.	Me llevo bien con mis compañeros de trabajo	1	2	3	4
K6.	Soy responsable para decidir cuánto trabajo hago en mi empleo	1	2	3	4
K7.	Mi trabajo requiere que haga las mismas cosas una y otra vez	1	2	3	4
K8.	Mi trabajo es frenético	1	2	3	4
K9.	Hay un buen ambiente en mi lugar de trabajo	1	2	3	4
K10.	Mi trabajo conlleva aprender nuevas cosas	1	2	3	4
K11.	Mi trabajo es exigente psicológicamente	1	2	3	4
K12.	Hay una buena cohesión en mi lugar de trabajo	1	2	3	4
K13.	Mi trabajo requiere que utilice un alto nivel de destrezas	1	2	3	4
K14.	En el trabajo hay a menudo conflictos y discusiones	1	2	3	4
K15.	Tengo control sobre cómo hago mi trabajo	1	2	3	4
